# Fibroblast radiosensitivity measured using the comet DNA-damage assay correlates with clonogenic survival parameters

**DOI:** 10.1038/sj.bjc.6690219

**Published:** 1999-03

**Authors:** A M Eastham, B Marples, A E Kiltie, C J Orton, C M L West

**Affiliations:** Cancer Research Campaign Section of Genome Damage and Repair, Paterson Institute for Cancer Research, Wilmslow Road, Manchester, M20 4BX, UK

**Keywords:** DNA damage, fibroblasts, comet assay, intrinsic radiosensitivity, predictive assays

## Abstract

A study was made of the neutral comet assay as a potential method for measuring normal cell radiosensitivity. Eleven fibroblast strains were studied comprising nine derived from vaginal biopsies from pretreatment cervical cancer patients and two strains from radiosensitive individuals. DNA double strand break (dsbs) dose–response curves for both initial and residual (20-h repair time) damage were obtained over the dose range 0–240 Gy, with slopes varying 3.2 and 8-fold respectively. Clonogenic cell survival parameters were available for all the cell strains following both high- and low-dose rate irradiation. There were no correlations between the dose–response slope of the initial level of DNA dsbs and parameters that mainly describe the initial portion of clonogenic radiation survival curves (SF _2_, α, *D̄-*). A significant correlation (*r* = –0.63, *P* = 0.04) was found between the extent of residual DNA dsbs and clonogenicity for all 11 fibroblast strains. The parameter showing the highest correlation with fibroblast cell killing (*D-*) for the nine normal fibroblasts alone was the ratio of initial/residual DNA dsb dose–response slope (*r* = 0.80, *P* = < 0.01). A significant correlation (*r* = –0.67, *P* = 0.03) with clonogenic radiosensitivity was also found for all 11 cell strains when using the ratio of initial/residual DNA dsb damage at a single dose of 180 Gy. This study shows that fibroblast radiosensitivity measured using the neutral comet assay correlates with clonogenic radiation survival parameters, and therefore may have potential value in predictive testing of normal tissue radiosensitivity. © 1999 Cancer Research Campaign


*British Journal of Cancer* (1999) **79**(9/10), 1366–1371
© 1999 Cancer Research Campaign
Article no. bjoc.1998.0219

### Fibroblast radiosensitivity measured using the comet DNA-damage assay correlates with clonogenic survival parameters

**AM Eastham, B Marples[1], AE Kiltie, CJ Orton and CML West**

Cancer Research Campaign Section of Genome Damage and Repair, Paterson Institute for Cancer Research, Christie Hospital (NHS) Trust, Wilmslow Road, Manchester M20 4BX, UK

**Summary** A study was made of the neutral comet assay as a potential method for measuring normal cell radiosensitivity. Eleven fibroblast strains were studied comprising nine derived from vaginal biopsies from pretreatment cervical cancer patients and two strains from radiosensitive individuals. DNA double strand break (dsbs) dose–response curves for both initial and residual (20-h repair time) damage were obtained over the dose range 0–240 Gy, with slopes varying 3.2 and 8-fold respectively. Clonogenic cell survival parameters were available for all the cell strains following both high- and low-dose rate irradiation. There were no correlations between the dose–response slope of the initial level of DNA dsbs and parameters that mainly describe the initial portion of clonogenic radiation survival curves ($SF_2$, $\alpha$, $\bar{D}$). A significant correlation ($r$ = –0.63, $P$ = 0.04) was found between the extent of residual DNA dsbs and clonogenicity for all 11 fibroblast strains. The parameter showing the highest correlation with fibroblast cell killing ($\bar{D}$) for the nine normal fibroblasts alone was the ratio of initial/residual DNA dsb dose–response slope ($r$ = 0.80, $P$ = < 0.01). A significant correlation ($r$ = –0.67, $P$ = 0.03) with clonogenic radiosensitivity was also found for all 11 cell strains when using the ratio of initial/residual DNA dsb damage at a single dose of 180 Gy. This study shows that fibroblast radiosensitivity measured using the neutral comet assay correlates with clonogenic radiation survival parameters, and therefore may have potential value in predictive testing of normal tissue radiosensitivity.

**Keywords:** DNA damage; fibroblasts; comet assay; intrinsic radiosensitivity; predictive assays

Intrinsic cellular radiosensitivity has been shown to be involved in determining the severity of normal tissue response to radiotherapy (Burnet et al, 1994). This has led to interest in the potential of measuring cellular radiosensitivity in patients prior to the commencement of radiotherapy, using the results to individualize treatment and either reduce morbidity or increase local control (MacKay et al, 1998). Recently, there have been several promising correlations reported between clonogenic fibroblast radiosensitivity and the severity of late normal tissue complications following radiotherapy (Brock et al, 1995; Burnet et al, 1996; Johansen et al, 1996). However, the clonogenic assays used in the latter studies are probably too slow to be of routine clinical use because they can sometimes require several weeks to obtain a result. There is, therefore, interest in studying other potentially more rapid assays of normal cell radiosensitivity.

The precise process(es) that define intrinsic radiosensitivity are unknown. However, it is generally accepted that DNA double strand breaks (dsbs) which remain unrepaired, or are misrepaired, are important lesions (Ward, 1994). Consequently, attention has focused on assays that measure radiation-induced DNA dsb damage as potential rapid predictive tests of normal cell radiosensitivity. There is evidence for a relationship, in fibroblasts, between residual radiation-induced DNA dsbs scored either by pulsed field (PFGE) or graded voltage (GVGE) gel electrophoresis and measures of radiosensitivity derived from clonogenic survival experiments (Wurm et al, 1994; Kiltie et al, 1997; Zhou et al, 1998).

Although successful, these cell population-based electrophoresis methods are not as suited to measuring radiation-induced DNA dsbs in clinical material as is the neutral comet assay (Olive et al, 1991). In particular, the neutral comet assay requires no pretreatment radioactive precursor to label the DNA, it can be performed with a small number of cells (in theory, only a few hundred) and a measure of the heterogeneity of DNA damage within the cell population is obtained as damage is assessed on a cell by cell basis. We have recently demonstrated the potential of the neutral comet assay technique as a surrogate assay for radiosensitivity in a large series of cervical carcinoma cell lines showing a correlation between the level of unrepaired DNA dsbs and clonogenic survival measured after 2 Gy ($SF_2$) (Marples et al, 1998). However, to our knowledge, no study has evaluated the potential of the neutral comet assay to measure DNA dsbs in a series of fibroblast strains and compared the results with clonogenic measures of radiosensitivity. Therefore, in this study, DNA dsbs were measured immediately after radiation treatment and 20 h later in 11 fibroblast strains. The initial and residual DNA dsb dose–response curves were then compared with radiosensitivity measured using a clonogenic assay.

*Received 14 April 1998*
*Received 24 July 1998*
*Accepted 4 August 1998*

*Correspondence to:* B Marples, Gray Laboratory Cancer Research Trust, PO Box 100, Mount Vernon Hospital, Northwood, Middlesex HA6 2JR, UK

### MATERIALS AND METHODS

### Cell culture

The normal fibroblast strains were derived from vaginal biopsies obtained from patients with carcinoma of the cervix at examination under anaesthetic before radical radiotherapy (Kiltie et al, 1997). Two cell strains known to be highly sensitive to the lethal effects of ionizing radiation were used as positive controls. An ataxia telangiectasia cell strain (AT1) was obtained from Dr C Arlett (University of Sussex, Brighton, UK) and a T(–) B(–) SCID cell strain (PH1) was kindly provided by Dr JP DeVillartay (Hopital Necker-Enfants Malades, Paris, France). The fibroblasts were grown as monolayers in minimum essential medium (MEM) supplemented with 15% fetal calf serum (Biowhittaker, Wokingham, UK), 2 mM glutamine, 100 U ml$^{-1}$ penicillin and 100 µg ml$^{-1}$ streptomycin. All media and supplements were obtained from Gibco BRL (Paisley, UK). Cultures were maintained at 37°C in a 5% carbon dioxide humidified incubator. Plastic tissue culture flasks (25 cm$^2$ Falcon) were seeded with $3 \times 10^5$ cells for initial damage experiments or with $2 \times 10^5$ cells in 12.5 cm$^2$ flasks for residual damage experiments, and used in plateau phase after 7 days growth. A parallel study with these 11 cell strains indicated that this culture regime ensured the cell population was in plateau phase with a low S-phase content, as assessed by flow cytometry analysis (<7%) (Kiltie et al, 1997).

### Clonogenic assay

Cells were irradiated using a $^{60}$Co γ-ray source with a dose rate of either 1.08 Gy min$^{-1}$ at room temperature (high-dose rate) or 0.011 Gy min$^{-1}$ at 37°C (low-dose rate). The procedures for carrying out the clonogenic assay experiments have been described in detail elsewhere (Kiltie et al, 1997). Either two (vaginal strains) or three (AT1 and PH1) replicate experiments were performed on each cell strain. Radiation survival curves were fitted using a linear quadratic equation [surviving fraction = exp-($\alpha$d + $\beta$d$^2$)] to obtain the measures $\alpha$ (initial slope) and $\bar{D}$ (mean inactivation dose, the integral of fitted curves in linear co-ordinates).

### Comet assay

Cells were irradiated using a $^{137}$Cs γ-ray source at a dose rate of 3.1 Gy min$^{-1}$. For initial DNA damage experiments, plateau phase cells were trypsinized, counted and 0.5 ml of an ice-cold suspension at $4 \times 10^4$ cells ml$^{-1}$ was irradiated with 60, 120, 180 or 240 Gy. The cells were then returned immediately on ice to prevent repair. An unirradiated control flask was included in all experiments. For measurements of residual DNA damage, the cells were irradiated as a monolayer, to the same doses, followed by reincubation at 37°C for 20 ± 2 h to allow for repair, and then processed identically to the samples used for initial dsb DNA damage. At the time of sampling, 0.5 ml of cells ($4 \times 10^4$ ml$^{-1}$) was mixed with 2.5 ml of 1% low gelling temperature agarose (Type VII, Sigma Chemical, St. Louis, USA) and 500 µl pipetted onto a slide precoated previously with 200 µl of agarose. Once the agarose had solidified, the slides were carefully placed in lysis buffer (0.5% SDS, 30 mM EDTA, pH 8.9) for 4 h at 50°C (Olive et al, 1991) and then removed, rinsed in 400 ml 0.5 × TBE (45 mM Tris, 1 mM EDTA, 45 mM boric acid, pH 8.5) to remove excess lysis buffer and placed in fresh 0.5 × TBE overnight. Slides were then electrophoresed for 25 min in 1150 ml fresh 0.5 × TBE at 0.6 V cm$^{-1}$ with an average current of 5.1 mA, rinsed in double distilled water (DDW) and stained with propidium iodide (PI; 2.5 µg ml$^{-1}$ in 0.1 M sodium chloride) for 0.5 h. Unbound stain was removed by washing with DDW. Alternatively, slides were dried without staining and stored until required. Rehydration of the slides was by addition of 1 ml DDW for 0.5 h, followed by 1 ml PI for 0.5 h.

### Comet analysis

Comets were analysed with a Leitz Diaplan fluorescent microscope at 200 × magnification using a Kinetic Imaging Komet system (Liverpool, UK) (Ashby et al, 1995). Comet images were selected randomly from the microscope field of view using a defined sequence of searching to ensure the same image was only scored once. The parameter used as an index of DNA damage was tail moment, which combines a measure of the length of the comet tail and the proportion of DNA to migrate into the tail (Olive and Banáth, 1993). The mean tail moment value was calculated by the imaging software from 50 comets measured for each dose point per individual experiment. For each cell line, three independent experiments (initial and residual damage) were performed. To obtain an overall mean tail moment value for the three separate experiments, the values obtained from the individual experiments were normalized against an internal control and then averaged. This mean value and the standard error of the mean were plotted, fitted by linear regression and the slopes of the dose–response curves calculated. For statistical analysis, correlations between measures of DNA dsb damage and repair and clonogenicity were obtained using the Pearson correlation.

### RESULTS

Dose–response curves for initial and residual radiation-induced DNA dsbs were obtained for all 11 fibroblast strains in three replicate experiments. Figure 1 illustrates typical tail moment data for one strain (SV357) and shows the magnitude of experimental

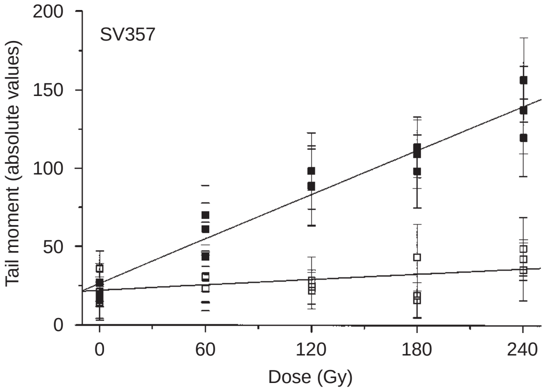

**Figure 1** Initial (■) and residual (□) DNA dsb dose–response curves calculated from absolute tail moment values for the SV357 fibroblast strain measured in three replicate experiments. Each point represents the mean (±s.d.) tail moment calculated from 50 cells

**Table 1** Slopes of the initial and residual DNA dsb dose–response curves. Ratio of slopes is calculated as initial damage/residual damage

| Cell strain | Initial damage slope | Residual damage slope | Ratio of slopes | Ratio at 180 Gy |
|---|---|---|---|---|
| AT1 | 0.026 ± 0.003 | 0.007 ± 0.001 | 3.8 ± 0.7 | 2.9 |
| PH1 | 0.012 ± 0.002 | 0.006 ± 0.001 | 2.0 ± 0.5 | 1.4 |
| SV269 | 0.031 ± 0.005 | 0.002 ± 0.002 | 19 ± 16 | 7.8 |
| SV282 | 0.026 ± 0.007 | 0.002 ± 0.0005 | 9.2 ± 4.7 | 5.0 |
| SV337 | 0.017 ± 0.003 | 0.001 ± 0.0004 | 19 ± 7.4 | 4.2 |
| SV350 | 0.014 ± 0.001 | 0.002 ± 0.0009 | 6.6 ± 3.1 | 2.8 |
| SV351 | 0.023 ± 0.002 | 0.002 ± 0.0005 | 12 ± 3.0 | 4.0 |
| SV357 | 0.023 ± 0.002 | 0.003 ± 0.002 | 8.1 ± 5.2 | 5.1 |
| SV368 | 0.024 ± 0.004 | 0.005 ± 0.003 | 4.9 ± 3.0 | 5.1 |
| SV371 | 0.039 ± 0.003 | 0.008 ± 0.004 | 5.1 ± 2.5 | 2.4 |
| SV372 | 0.027 ± 0.002 | 0.006 ± 0.002 | 4.5 ± 1.5 | 2.8 |

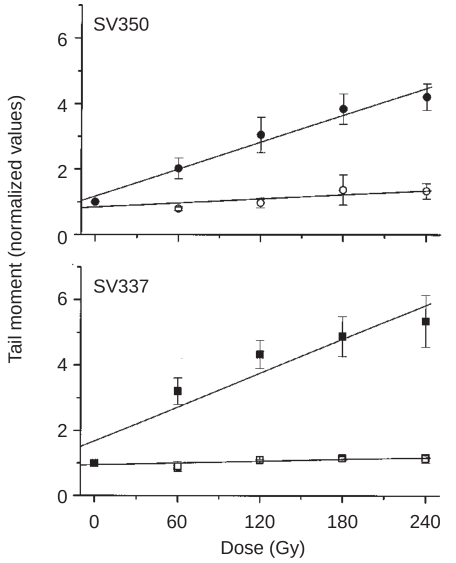

**Figure 2** Comparison of normalized initial (■) and residual (□) DNA dsb dose–response curves for 2 of the 11 cell strains studied. Each point represents the mean (±s.e.m.) of the normalized DNA dsb data calculated from three independent experiments

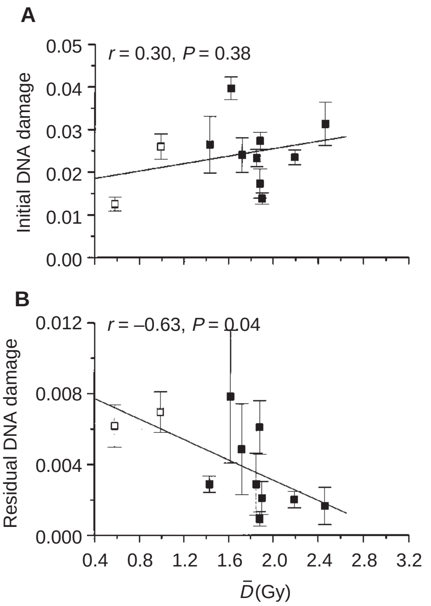

**Figure 3** Relationship between clonogenic radiosensitivity (represented by $\bar{D}$) and the slopes of initial (**A**) and residual (**B**) DNA dsb damage.

variation seen between the individual experiments. To compare directly the 11 cell strains, the data for each cell strain were normalized using the tail moment value obtained for unirradiated cells in each experiment. Figure 2 shows the results of normalization in two of the cell strains (SV350 and SV337). A clear difference is evident between the slopes of the initial and residual response curves, indicating repair of the induced DNA dsbs during the 20 h period that cells were held at 37°C after treatment.

The slopes of the DNA dsb dose–response curves from the combined data for each of the 11 fibroblast strains were calculated using linear regression, and these are shown in Table 1. These slope values ranged from 0.012 to 0.039 (3.2-fold) for initial DNA dsbs and from 0.001 to 0.008 (8-fold) for residual DNA dsbs. Statistical comparison (non-parametric one way analysis of variance) between the initial ($P$ = 0.16) and residual ($P$ = 0.06) slope values obtained from the individual experiments indicate no significant difference between the 11 cell strains. However, the slopes of the individual residual damage dose–response curves could be used to discriminate between the two radiosensitive (AT and PH1) and nine normal cell strains (Mann–Whitney $U$-test: $P$ = 0.01). No discrimination was found for initial damage ($P$ = 0.11).

For each cell line, the ratio of the dose–response slopes was also calculated by dividing values for the initial slopes by those for the residual slopes. The ratio parameter is an index of DNA damage repair capacity and the values ranged from 2 to 19 (i.e. a 9.5-fold variation between all 11 fibroblasts with a 4-fold variation between the nine normal fibroblast strains) (Table 1).

Clonogenic radiation survival curves and parameters have been published elsewhere for the two radiosensitive strains (Sproston et al, 1997) and the nine normal strains (Kiltie et al, 1997). A brief

 © Cancer Research Campaign 1999

**Table 2** Radiation survival parameters

| Cell strain | $SF_2$ | | α ($Gy^{-1}$) | | $\bar{D}$ (Gy) | |
|---|---|---|---|---|---|---|
| | HDR | LDR | HDR | LDR | HDR | LDR |
| AT1 | 0.03 ± 0.002 | 0.06 ± 0.05 | 1.19 ± 0.59 | 0.90 ± 0.23 | 0.71 | 0.99 |
| PH1 | 0.03 ± 0.004 | 0.03[a] | 1.68 ± 0.04 | 1.71 ± 0.10 | 0.60 | 0.58 |
| SV269 | 0.21 ± 0.01 | 0.29 ± 0.03 | 0.52 ± 0.07 | 0.21 ± 0.07 | 1.62 | 2.46 |
| SV282 | 0.18 ± 0.01 | 0.23 ± 0.01 | 0.84 ± 0.05 | 0.80 ± 0.04 | 1.20 | 1.43 |
| SV337 | 0.32 ± 0.02 | 0.30 ± 0.01 | 0.30 ± 0.07 | 0.33 ± 0.07 | 1.80 | 1.88 |
| SV350 | 0.15 ± 0.01 | 0.35 ± 0.06 | 0.96 ± 0.06 | 0.56 ± 0.03 | 1.13 | 1.90 |
| SV351 | 0.19 ± 0.01 | 0.31 ± 0.01 | 0.71 ± 0.06 | 0.33 ± 0.05 | 1.25 | 2.19 |
| SV357 | 0.18 ± 0.01 | 0.33 ± 0.03 | 0.75 ± 0.04 | 0.54 ± 0.04 | 1.32 | 1.85 |
| SV368 | 0.18 ± 0.01 | 0.31 ± 0.02 | 0.83 ± 0.08 | 0.51 ± 0.04 | 1.14 | 1.72 |
| SV371 | 0.21 ± 0.01 | 0.24 ± 0.02 | 0.75 ± 0.05 | 0.59 ± 0.05 | 1.26 | 1.62 |
| SV372 | 0.29 ± 0.02 | 0.33 ± 0.02 | 0.64 ± 0.04 | 0.48 ± 0.04 | 1.42 | 1.88 |

[a] Only one experiment performed for PH1. Values are means and standard errors of two or three independent experiments.

**Table 3** Correlations of survival parameters with repair capacity using the Pearson correlation

| | Initial slopes (all strains) | | Residual slopes (all strains) | | Ratio of slopes (all strains) | | Ratio of slopes (SV strains only) | | Ratio at 180 Gy (all strains) | | Ratio at 180 Gy (SV strains only) | |
|---|---|---|---|---|---|---|---|---|---|---|---|---|
| | *r* | *P* | *r* | *P* | *r* | *P* | *r* | *P* | *r* | *P* | *r* | *P* |
| **HDR $SF_2$** | 0.25 | 0.46 | –0.42 | 0.20 | 0.58[a] | 0.06 | 0.38 | 0.31 | 0.35 | 0.29 | –0.13 | 0.74 |
| **LDR $SF_2$** | 0.13 | 0.71 | –0.55[a] | 0.08 | 0.41 | 0.21 | –0.14 | 0.72 | 0.45 | 0.17 | –0.15 | 0.69 |
| **HDR** α | –0.39 | 0.24 | 0.53[a] | 0.10 | –0.74[b] | 0.01 | –0.76[b] | 0.02 | –0.58[a] | 0.06 | –0.28 | 0.47 |
| **LDR** α | –0.40 | 0.22 | 0.52[a] | 0.10 | –0.64[b] | 0.03 | –0.68[b] | 0.04 | –0.62[b] | 0.04 | –0.40 | 0.29 |
| **HDR** $\bar{D}$ | 0.24 | 0.43 | –0.6[b] | 0.05 | 0.80[c] | 0.003 | 0.80[c] | 0.009 | 0.59[a] | 0.06 | 0.34 | 0.37 |
| **LDR** $\bar{D}$ | 0.30 | 0.38 | –0.63[b] | 0.04 | 0.68[b] | 0.02 | 0.60[a] | 0.09 | 0.67[b] | 0.03 | 0.48 | 0.19 |
| **PFGE** | –0.37 | 0.27 | 0.54[a] | 0.08 | –0.63[b] | 0.02 | –0.62[a] | 0.08 | –0.62[b] | 0.04 | –0.41 | 0.28 |

[a] Correlation is significant at the 0.10 level (two-tailed). [b] Correlation is significant at the 0.05 level (two-tailed). [c] Correlation is significant at the 0.01 level (two-tailed).

summary of the data is given in Table 2. The high-dose rate $SF_2$ values ranged from 0.028 to 0.32, differing by a factor of 12. A comparison was made between the clonogenic survival parameters and the measures of DNA damage. No correlation was seen for clonogenicity and initial damage (Figure 3). However, a significant correlation was evident when clonogenic radiosensitivity was compared with residual DNA dsb damage (Figure 3). Moreover, significant correlations were seen when the ratio of the initial/residual DNA dsb slopes were compared with the various survival parameters (Table 3). Figure 4 illustrates the strongest correlation which was found between the ratio of slopes and high-dose rate $\bar{D}$ when the two radiosensitive strains were either included ($r$ = 0.80, $P$=0.003) or excluded ($r$ = 0.80, $P$ = 0.009). Statistical analysis of the clonogenicity values in combination with either the meaned initial (Mann–Whitney $U$-test: $P$ = 0.44) or residual damage ($P$ = 0.22) data in Figure 3 indicate no discrimination between the two radiosensitive (AT and PH1) and nine normal cell strains. However, clonogenicity combined with the ratio parameter of initial/residual slopes in Figure 4 was found to discriminate between the radiosensitive and normal fibroblasts strains ($P$ = 0.04).

Correlations were then examined using the ratio of the absolute level of initial and residual DNA dsbs at a single dose point (180 Gy). No significant correlations were evident when the radiosensitive syndromic cell strains (AT1 and PH1) were excluded. However, when all 11 fibroblast strains were included, correlations of borderline significance were found (Table 3). The strongest correlation found was between the ratio of DNA dsb damage at 180 Gy and radiosensitivity (low-dose rate $\bar{D}$) ($r$ = 0.67 $P$ = 0.03) is shown in Figure 5.

### DISCUSSION

In this study, DNA dsb induction and repair were measured in nine normal and two radiosensitive fibroblast cell strains. To compare the data from all the 11 fibroblast cell strains, comet tail moment values for the individual cell strains required normalization against the unirradiated controls from within each experiment. This normalization procedure was necessary to accommodate the variation in comet-head size of unirradiated cells between the different fibroblast cell strains. The reason for this variation is unknown, but the rigid experimental protocols used for growing the cells and

© Cancer Research Campaign 1999

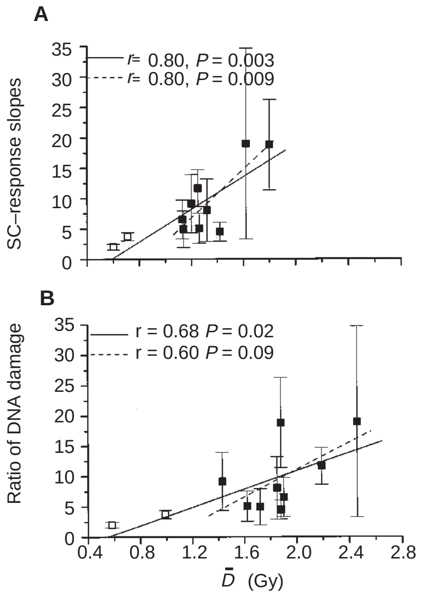

**Figure 4** Relationship between clonogenic radiosensitivity (represented by $\bar{D}$) at high (**A**) and low (**B**) dose rates and the ratio of DNA dsb damage slopes for all the cell strains; nine normal fibroblast strains (■) and the sensitive cell strains AT1 and PH1 (□). The solid line indicates the fit through all 11 strains and the dashed line indicates the fit through the nine normal fibroblast strains

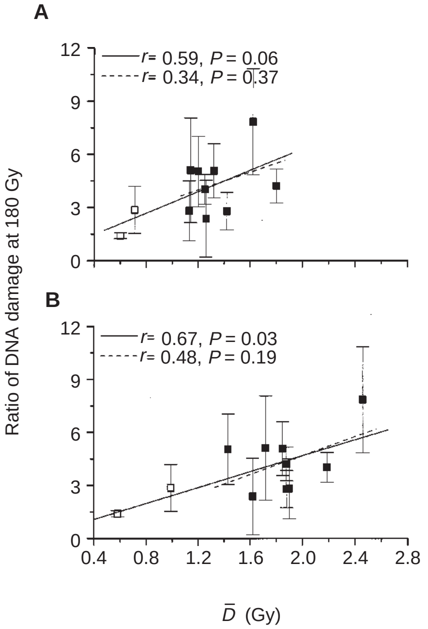

**Figure 5** Relationship between clonogenic radiosensitivity (represented by $\bar{D}$) at high (**A**) and low (**B**) dose rates and the ratio of DNA damage at a single dose point of 180 Gy for all the cell strains, including the sensitive cell strains AT1 and PH1 (□). The solid line indicates the fit through all 11 strains and the dashed line indicates the fit through the nine normal fibroblast (■) strains measuring DNA breaks would suggest it does not reflect experimental variability between the individual experiments (e.g. cell cycle difference, lysis conditions, PI staining intensity). Rather, it appears to be a trait of untransformed fibroblasts and may reflect the differentiation status (Rodemann and Bamberg, 1995) of each strain (e.g. differential sensitivity to the lysis conditions) because tail moment normalization for the above reason was not found to be necessary in a previous study in our laboratory measuring DNA damage in a range of cervical tumour cell lines (Marples et al, 1998).

The dose range used here was chosen after preliminary experiments (data not shown) had indicated that the tail moment values reached a plateau at doses > 240 Gy. This is not unexpected because the comet tail consists of broken strands of DNA attached to the nuclear matrix plus damaged stretched loops of DNA, and it is therefore finite in length (Fairbairn et al, 1995). Consequently, over the dose range used in this study, linear initial and residual DNA dsb dose–response curves were seen for all the cell strains, which is in agreement with other neutral comet assay studies in human cells (Olive et al, 1994: Marples et al, 1998). Studies measuring radiation-induced DNA damage using PFGE have reported non-linear initial (Wurm et al, 1994) and residual (Kiltie et al, 1997) dose responses in fibroblast strains. Analyses of data in the latter cases were restricted to the linear portion of these dose–response curves, in agreement with this report and other published data (Iliakis et al, 1991).

Analysis of variation of initial DNA dsb damage slopes showed no significant differences between all the 11 strains ($P = 0.10$) and the nine vaginal fibroblast strains ($P = 0.21$), and a non-significant correlation with clonogenicity (Figure 3). The lack of significant variation in the slopes of the initial DNA damage dose–response curves is in agreement with PFGE and CHEF data obtained by Blocher et al (1991) and Hannan et al (1991), respectively, but contrasts with those of others obtained by PFGE (Wurm et al, 1994).

Previous studies using fibroblasts which have demonstrated a correlation between residual DNA damage and clonogenic measurements of radiosensitivity have used a 4 h repair period (Wurm et al, 1994; Zhou et al, 1998). Because Badie et al (1995) reported that residual damage measured at time intervals longer than 4 h was likely to be a better discriminator of DNA repair levels in fibroblasts, therefore we chose to use an overnight (20 h) repair period in this study. This decision was endorsed by the recent data of Kiltie et al (1997), which demonstrated an excellent correlation between a measure of clonogenic radiosensitivity and the extent of residual DNA damage 24 h after treatment measured by PFGE in the same fibroblast strains used in the present study. For all 11 fibroblast strains studied here, the residual damage dose–response curves were less steep than those for initial damage, indicating that the majority of the repairable radiation-induced DNA dsb damage was indeed repaired during the 20 h incubation period.

The slopes of the residual DNA dsb dose–response curves for the nine normal fibroblasts varied by a factor of 8, four times that of the initial damage slopes (Table 1). A difference of borderline significance was seen in the level (slopes) of residual DNA dsbs between the 11 cell strains ($P = 0.06$). Moreover, discrimination was seen between the level of residual damage between the radiosensitive strains and normal strains ($P = 0.01$).

A significant correlation was seen between the extent of residual DNA dsbs measured after 20 h using the neutral comet assay and clonogenic radiosensitivity for all 11 fibroblast strains ($r = –0.63$, $P = 0.04$) (Figure 3). This is in agreement with studies that have shown correlations between cell kill and the extent of DNA dsb repair as assessed by PFGE in fibroblast strains at 4 and 24 h after

 © Cancer Research Campaign 1999

irradiation (Zhou et al, 1998; Wurm et al, 1994; Kiltie et al, 1997). In this comet study, stronger relationships were seen when the ratio of initial and residual DNA damage over 0–240 Gy dose range, reflecting the proportion of DNA dsbs unrepaired, was compared with a measure of clonogenic radiosensitivity for the nine normal and all 11 fibroblasts (Table 3, Figure 4). Clearly, these data indicate the potential of residual DNA dsbs and/or the ratio of initial/residual breaks, as measured by the neutral comet assay, as a method of predicting the radiosensitivity of fibroblasts. However, for a predictive assay of radiosensitivity to be adopted clinically, it must also be rapid and simple. We, therefore, compared the absolute level of DNA dsbs assessed at a single dose of 180 Gy with clonogenic radiosensitivity. However, significant correlations were found only when the data from all 11 fibroblasts were compared with clonogenicity, and non-significant trends were seen for the nine normal fibroblasts (Table 3, Figure 5).

For a novel rapid assay to be beneficial for predictive testing, a clear discrimination must be detected between radiosensitive (e.g. AT1 and PH1) and normal cell strains because clear differences are evident in the clonogenicity data (Sproston et al, 1997; Kiltie et al, 1997). Statistically significant discrimination was detected only between the two radiosensitive strains and normal fibroblasts in this study when comparing the ratio of initial and residual damage slopes with $\bar{D}$ ($P = 0.04$), supporting the use of this ratio parameter in predictive testing of clonogenicity using the neutral comet assay.

A comparable study on the same fibroblast cell strains as used here, but assessing DNA dsbs using the PFGE assay, also found correlations between residual damage at 24 h and clonogenicity data (Kiltie et al, 1997). The PFGE assay correlations were stronger than those reported here, possibly indicating that the PFGE results give a better prediction of radiosensitivity than those from the neutral comet assay. In addition, strong significant correlation was also reported after a single dose of 150 Gy in the PFGE study, in contrast to the comet data reported here.

In summary, this study has shown that there is a relationship between neutral comet assay results and clonogenic measurements of normal cell radiosensitivity. The ratio of initial and residual DNA dsb damage, which probably represents the fraction of DNA damage remaining after a period of repair, was found to be the best measure of radiation-induced DNA damage relating to cellular radiosensitivity.

### ACKNOWLEDGEMENTS

Discussions with Professor Jolyon Hendry and Dr David Scott are gratefully acknowledged. The authors particularly thank Dr Steve Roberts for help with data analysis. This work was supported by the Christie Hospital Endowment Fund and the Cancer Research Campaign, UK.

### REFERENCES

Ashby J, Tinwell H, Lefevre PA and Browne MA (1995) The single cell gel electrophoresis assay for induced DNA damage (comet assay): measurement of tail length and moment. *Mutagenesis* **10**: 85–90

Badie C, Iliakis G, Foray N, Alsbeih G, Cedervall B, Chavaudra N, Pantelias G, Arlett C and Malaise EP (1995) Induction and rejoining of DNA double-strand breaks and interphase chromosome breaks after exposure to X rays in one normal and two hypersensitive human fibroblast cell lines. *Radiat Res* **144**: 26–35

Blocher D, Sigut D and Hannan MA (1991) Fibroblasts from ataxia telangiectasia (AT) and AT heterozygotes show an enhanced level of residual DNA double-strand breaks after low dose-rate gamma-irradiation as assayed by pulsed field gel electrophoresis. *Int J Radiat Biol* **60**: 791–802

Brock WA, Tucker SL, Geara FB, Turesson I, Wike J, Nyman J and Peters LJ (1995) Fibroblast radiosensitivity versus acute and late normal skin responses in patients treated for breast cancer. *Int J Radiat Oncol Biol Phys* **32**: 1371–1379

Burnet NG, Nyman J, Turesson I, Wurm R, Yarnold JR and Peacock JH (1994) The relationship between cellular radiation sensitivity and tissue response may provide the basis for individualising radiotherapy schedules. *Radiother Oncol* **33**: 228–238

Burnet NG, Wurm R and Peacock JH (1996) Low dose-rate fibroblast radiosensitivity and the prediction of patient response to radiotherapy. *Int J Radiat Biol* **70**: 289–300

Fairbairn DW, Olive PL and O'Neill KL (1995) The comet assay: a comprehensive review. *Mutat Res* **339**: 37–59

Hannan MA, Blocher D, Sigut D, Waghray M (1991) DNA double strand breaks in fibroblast cell lines, from non-Hodgkin's lymphoma patients, showing increased sensitivity to chronic gamma irradiation. *Cancer Lett* **57**: 137–143

Iliakis GE, Metzger L, Denko N and Stamato TD (1991) Detection of DNA double-strand breaks in synchronous cultures of CHO cells by means of asymmetric field inversion gel electrophoresis. *Int J Radiat Biol* **59**: 321–341

Johansen J, Bentzen SM, Overgaard J and Overgaard M (1996) Relationship between the in vitro radiosensitivity of skin fibroblasts and the expression of subcutaneous fibrosis, telangiectasia, and skin erythema after radiotherapy. *Radiother Oncol* **40**: 101–109

Kiltie AE, Orton CJ, Ryan A, Roberts SA, Marples B, Davidson SE, Hunter RD, Margison G, West CML and Hendry JH (1997). A correlation between DNA damage and clonogenic measurements of radiosensitivity in fibroblasts from pre-radiotherapy cervix cancer patients. *Int J Radiat Oncol Biol Phys* **39**: 1137–1144

MacKay RI, Niemierko A, Goitein M, Hendry JH (1998) Potential clinical impact of normal tissue intrinsic radiosenstivity testing. *Radiother Oncol* **46**: 215–216

Marples B, Longhurst D, Eastham AM and West CML (1998) The ratio of initial/residual DNA damage predicts intrinsic radiosensitivity in 7 cervix carcinoma cell lines. *Br J Cancer* **77**: 1108–1114

Olive PL and Banáth JP (1993) Induction and rejoining of radiation induced DNA single-strand breaks: tail moment as a function of position in the cell cycle. *Mutat Res* (DNA repair) **294**: 275–283

Olive PL, Wlodek D and Banáth JP (1991) DNA double strand breaks measured in individual cells subjected to gel electrophoresis. *Cancer Res* **51**: 4671–4676

Olive PL, Banáth JP and MacPhail SM (1994) Lack of a correlation between radiosensitivity and DNA double-strand break induction or rejoining in six human tumour cell lines. *Cancer Res* **54**: 3939–3946

Rodemann HP and Bamberg M (1995) Cellular basis of radiation-induced fibrosis. *Radiother Oncol* **35**: 83–90

Sproston ARM, West CML and Hendry JH (1997) Cellular radiosensitivity in human severe-combined-immunodeficiency (SCID) syndromes. *Radiother Oncol* **42**: 53–57

Ward JF (1994) The complexity of DNA damage: relevance to biological consequences. *Int J Radiat Biol* **66**: 427–432

Wurm R, Burnet NG, Duggal N, Yarnold JR and Peacock JH (1994) Cellular radiosensitivity and DNA damage in primary human fibroblasts. *Int J Radiat Oncol Biol Phys* **30**: 625–633

Zhou PK, Sproston ARM, Marples B, West CML, Margison GP and Hendry JH (1998) The radiosensitivity of human fibroblast cell lines correlates with residual levels of DNA double-strand breaks. *Radiother Oncol* **47**: 271–276

© Cancer Research Campaign 1999

## References

[PDF_00536] Ashby J., Tinwell H., Lefevre P. A., Browne M. A. (1995). The single cell gel electrophoresis assay for induced DNA damage (comet assay): measurement of tail length and moment.. Mutagenesis.

[PDF_00539] Badie C., Iliakis G., Foray N., Alsbeih G., Cedervall B., Chavaudra N., Pantelias G., Arlett C., Malaise E. P. (1995). Induction and rejoining of DNA double-strand breaks and interphase chromosome breaks after exposure to X rays in one normal and two hypersensitive human fibroblast cell lines.. Radiat Res.

[PDF_00544] Blöcher D., Sigut D., Hannan M. A. (1991). Fibroblasts from ataxia telangiectasia (AT) and AT heterozygotes show an enhanced level of residual DNA double-strand breaks after low dose-rate gamma-irradiation as assayed by pulsed field gel electrophoresis.. Int J Radiat Biol.

[PDF_00548] Brock W. A., Tucker S. L., Geara F. B., Turesson I., Wike J., Nyman J., Peters L. J. (1995). Fibroblast radiosensitivity versus acute and late normal skin responses in patients treated for breast cancer.. Int J Radiat Oncol Biol Phys.

[PDF_00552] Burnet N. G., Nyman J., Turesson I., Wurm R., Yarnold J. R., Peacock J. H. (1994). The relationship between cellular radiation sensitivity and tissue response may provide the basis for individualising radiotherapy schedules.. Radiother Oncol.

[PDF_00556] Burnet N. G., Wurm R., Peacock J. H. (1996). Low dose-rate fibroblast radiosensitivity and the prediction of patient response to radiotherapy.. Int J Radiat Biol.

[PDF_00559] Fairbairn D. W., Olive P. L., O'Neill K. L. (1995). The comet assay: a comprehensive review.. Mutat Res.

[PDF_00561] Hannan M. A., Blöcher D., Sigut D., Waghray M. (1991). DNA double strand breaks in fibroblast cell lines, from non-Hodgkin's lymphoma patients, showing increased sensitivity to chronic gamma irradiation.. Cancer Lett.

[PDF_00564] Iliakis G. E., Metzger L., Denko N., Stamato T. D. (1991). Detection of DNA double-strand breaks in synchronous cultures of CHO cells by means of asymmetric field inversion gel electrophoresis.. Int J Radiat Biol.

[PDF_00567] Johansen J., Bentzen S. M., Overgaard J., Overgaard M. (1996). Relationship between the in vitro radiosensitivity of skin fibroblasts and the expression of subcutaneous fibrosis, telangiectasia, and skin erythema after radiotherapy.. Radiother Oncol.

[PDF_00571] Kiltie A. E., Orton C. J., Ryan A. J., Roberts S. A., Marples B., Davidson S. E., Hunter R. D., Margison G. P., West C. M., Hendry J. H. (1997). A correlation between residual DNA double-strand breaks and clonogenic measurements of radiosensitivity in fibroblasts from preradiotherapy cervix cancer patients.. Int J Radiat Oncol Biol Phys.

[PDF_00576] MacKay R. I., Niemierko A., Goitein M., Hendry J. H. (1998). Potential clinical impact of normal-tissue intrinsic radiosensitivity testing.. Radiother Oncol.

[PDF_00578] Marples B., Longhurst D., Eastham A. M., West C. M. (1998). The ratio of initial/residual DNA damage predicts intrinsic radiosensitivity in seven cervix carcinoma cell lines.. Br J Cancer.

[PDF_00581] Olive P. L., Banáth J. P. (1993). Induction and rejoining of radiation-induced DNA single-strand breaks: "tail moment" as a function of position in the cell cycle.. Mutat Res.

[PDF_00587] Olive P. L., Banáth J. P., MacPhail H. S. (1994). Lack of a correlation between radiosensitivity and DNA double-strand break induction or rejoining in six human tumor cell lines.. Cancer Res.

[PDF_00584] Olive P. L., Wlodek D., Banáth J. P. (1991). DNA double-strand breaks measured in individual cells subjected to gel electrophoresis.. Cancer Res.

[PDF_00590] Rodemann H. P., Bamberg M. (1995). Cellular basis of radiation-induced fibrosis.. Radiother Oncol.

[PDF_00592] Sproston A. R., West C. M., Hendry J. H. (1997). Cellular radiosensitivity in human severe-combined-immunodeficiency (SCID) syndromes.. Radiother Oncol.

[PDF_00595] Ward J. F. (1994). The complexity of DNA damage: relevance to biological consequences.. Int J Radiat Biol.

[PDF_00597] Wurm R., Burnet N. G., Duggal N., Yarnold J. R., Peacock J. H. (1994). Cellular radiosensitivity and DNA damage in primary human fibroblasts.. Int J Radiat Oncol Biol Phys.

[PDF_00600] Zhou P. K., Sproston A. R., Marples B., West C. M., Margison G. P., Hendry J. H. (1998). The radiosensitivity of human fibroblast cell lines correlates with residual levels of DNA double-strand breaks.. Radiother Oncol.

